# Automated precise recognition of ovine embryo microscopic images using a boundary-enhanced deep learning network

**DOI:** 10.1262/jrd.2026-024

**Published:** 2026-06-21

**Authors:** Zhihui SHI, Chongchong YU, Jingyu REN, Jie ZHU, Dongyue ZHOU, Haixia WANG, Mingyue YANG, Hanqing YANG

**Affiliations:** 1) School of Computer and Artificial Intelligence, Beijing Technology and Business University, Beijing 102488, China; 2) Beijing Jingwa Agricultural Science & Technology Innovation Center, Beijing 101205, China; 3) Key Laboratory of Herbivorous Livestock Reproductive Regulation, National Sheep Genetic Evaluation Center, Inner Mongolia University, Hohhot 010030, China

**Keywords:** Deep learning, *In vitro* fertilization, Microscopy, Ovine embryo

## Abstract

Efficient selection of *in vitro*-fertilized embryos is crucial for assisted reproduction in sheep, and automated microscopic analysis can enable objective evaluation. Instance segmentation is a key prerequisite for extracting downstream morphological and morphokinetic indicators. However, ovine embryo microscopic images typically exhibit abundant cytoplasmic lipid droplets and tightly packed blastomeres, causing conventional segmentation methods to fail when cell boundaries are faint. Here, we developed a boundary-enhanced deep learning network (BE-DFNet) to address high-density cell adhesion. By introducing a boundary stream as a structural prior to focus on cell–cell contact interfaces, the model was validated on 847 2D brightfield images covering the early cleavage stages (from 1-cell to 16-cell). We specifically focused on these stages because they provide critical morphokinetic indicators (e.g., t2 to t8) before significant blastomere overlapping in the compacted morula stage physically limits the reliability of 2D instance segmentation. BE-DFNet achieved a panoptic quality of 0.5088, outperforming a representative state-of-the-art method, Cellpose-SAM (0.4323). Notably, BE-DFNet reduced endpoint error, which reflects topological consistency, from 1.6265 to 0.4632, indicating improved separation of adhered blastomeres. Collectively, BE-DFNet effectively mitigates cytoplasmic texture interference and ensures the high-precision topological reconstruction of blastomere boundaries, providing a reliable foundation for fully automated morphokinetic grading.

***I****n vitro* fertilization (IVF) and embryo transfer (ET) have become key assisted reproductive technologies (ART) for improving genetic gain and reproductive efficiency in modern livestock production [1, 2]. In sheep, these technologies are used not only for commercial breeding but also to support their role as an important large-animal model for reproductive medicine research [3]. Although the production of IVF embryos has increased annually, pregnancy rates after ET are often lower for IVF-derived embryos than for embryos produced *in vivo* [4, 5]. Therefore, accurate selection of embryos with high developmental potential is essential to establish pregnancy [6].

At present, the morphological grading system established by the International Embryo Technology Society (IETS) is regarded as the gold standard for evaluating bovine and ovine embryo quality [7]. However, this single-time-point, morphology-based assessment is subjective and highly dependent on embryologists’ experience, which can lead to inconsistent evaluations [8, 9]. In recent years, visible-light time-lapse monitoring (TLM) has emerged as a powerful noninvasive tool. TLM enables continuous observation of embryo development and captures morphokinetic parameters that may be missed by static observation, such as cleavage timing (t2, t4, t8), blastomere number, and symmetry of cell division [10,11,12]. These parameters are closely associated with blastocyst formation and post-transfer pregnancy success [13, 14]. Accordingly, stable and accurate extraction of cleavage events and quantitative morphological indicators from ovine embryo image sequences is a fundamental step toward automated and reproducible evaluation [15]. Instance-level segmentation of blastomeres is central to cell counting, morphological measurement, and automated recognition.

Although deep learning-based instance segmentation models have shown strong potential in human and mouse embryos [16, 17], applying them directly to ovine embryos remains challenging. Compared with human embryos, ovine embryos contain dense cytoplasmic lipid droplets, producing pronounced texture noise and a darker cytoplasmic appearance [18]. Such high-density texture can obscure physical boundaries between cells, making existing segmentation networks (e.g., Cellpose-SAM) prone to incorrectly merging adjacent, tightly stacked blastomeres (instance adhesion) [19, 20]. These topological errors directly bias cell counting and can mislead developmental stage determination and downstream grading.

To address these issues, we developed a Boundary-Enhanced Dual-flow Fusion Network (BE-DFNet) for brightfield time-series images of ovine embryos during cleavage stages. Unlike conventional models (e.g., Cellpose-SAM) that primarily rely on single-stream global features and often struggle to distinguish dense cytoplasmic lipid droplets from true cell borders, BE-DFNet utilizes a unique dual-stream architecture. Its key distinction lies in incorporating a dedicated boundary stream as a structural prior to explicitly extract high-frequency cell-cell contact interfaces. By asymmetrically fusing these boundary cues with main-stream semantic features, BE-DFNet effectively suppresses texture noise and improves separability and topological consistency in closely contacting blastomeres. We trained and validated the method on a self-built dataset of ovine embryo time-lapse microscopy images, providing an image-analysis basis for objective and reproducible morphokinetic grading.

## Materials and Methods

### Ethics statement

All animal experimental procedures were performed in strict accordance with guidelines approved by the Animal Care and Use Committee of Inner Mongolia University. Ovine ovaries were collected from a slaughterhouse for commercial meat production, with no additional handling of live animals. Semen was obtained from commercially available frozen semen.

### Collection of ovaries and in vitro embryo production

Ovine ovaries were obtained from a local slaughterhouse and transported to the laboratory within 2 h in sterile saline maintained at 30–35°C, supplemented with 100 IU/ml penicillin and 100 IU/ml streptomycin (P/S, 15140122, Thermo Fisher, Shanghai, China). Cumulus–oocyte complexes (COCs) were aspirated from follicles (2–8 mm in diameter) using an 18-gauge needle. COCs with homogeneous cytoplasm and surrounded by compact cumulus cell layers were selected. Briefly, COCs were cultured in groups of 40–50 in 500 ml of TCM-199 medium (11150067, Thermo Fisher) supplemented with 10% fetal bovine serum (FBS, 30044333, Thermo Fisher), 1 mg/ml estradiol (E8875, Sigma-Aldrich, Shanghai, China), 0.5 mg/ml FSH (Sansheng, Ningbo, China), and 0.1 mM cysteine (M9768, Sigma-Aldrich) for 22–24 h at 38.5°C in 5% CO_2_. Following maturation, cumulus cells were removed from the COCs by incubation in TCM-199 medium containing 0.1% hyaluronidase (H3506, Sigma-Aldrich) and gentle pipetting. Denuded MII oocytes with a visible first polar body and uniform cytoplasm were selected for subsequent experiments.

Before IVF, the denuded oocytes were transferred into the fertilization medium as 500 ml drops of SOF medium (synthetic oviduct fluid medium, self-prepared) supplemented with 2% oestrus ovine serum, 2 mM sodium pyruvate (P2256, Sigma-Aldrich), 10 mg/ml heparin sodium (H3149, Sigma-Aldrich) and 1 mM caffeine (58082, Amresco, Shanghai, China) in a 4-well culture dish and cultured in a CO_2_ incubator (38.5°C, 5% O_2_ and 5% CO_2_). For IVF, frozen semen straws (LEKE, Inner Mongolia, China) were removed from the liquid nitrogen, kept in air for 10 sec and thawed in a water bath at 38°C for 15 sec. After centrifugation with 90% and 45% Percoll solution (P1644, Sigma-Aldrich) diluted with the fertilization medium at 500 × *g* for 15 min, the spermatozoa were collected from the bottom of the tube. The spermatozoa at a final concentration of approximately 1 × 10^6^ sperm/ml were transferred to the fertilization medium containing MII oocytes and cultured for 18 h (38.5°C, 5% O_2_ and 5% CO_2_).

Fertilized embryos were then transferred to synthetic oviduct fluid containing amino acids (SOFaa) supplemented with 3 mg/ml bovine serum albumin (BSA, A1933, Sigma-Aldrich) and cultured at 38.5°C under 5% CO_2_, 5% O_2_.

### Image acquisition

Static microscopic imaging: To build a high-precision baseline dataset for training, embryos at different developmental stages were imaged using an inverted microscope equipped with a high-resolution CCD camera (Nikon Eclipse Ti2-U; Nikon, Tokyo, Japan) at 200 × or 400 × magnification. The focal plane was adjusted to the equatorial section to maximize visibility of the blastocoel and cell boundaries. Images were saved in uncompressed TIFF format (2048 × 2048 pixels) to preserve fine morphological texture for subsequent AI grading annotation according to the IETS manual (5th edition).

Continuous morphokinetic imaging: To monitor full developmental dynamics from fertilization to the blastocyst stage, continuous imaging was performed using an Agilent Cytation 7 cell imaging multimode reader (Agilent Technologies, Santa Clara, CA, USA). Following 18 h of co-incubation (Day 0), presumptive zygotes were further denuded of remaining cumulus cells and adherent spermatozoa by gentle pipetting in 0.1% hyaluronidase to ensure clear blastomere boundaries for accurate instance segmentation. The denuded embryos in groups were then cultured in 4-well culture dish containing SOFaa medium under mineral oil (M8410; Sigma-Aldrich). The imaging system’s incubation chamber maintained environmental conditions (38.5ºC, 5% CO_2_, and 5% O_2_) consistent with the standard incubator settings.

Automated imaging commenced at 18 h post-insemination (hpi), Images were acquired in brightfield mode every 120 min for 168 h (7 days). This sampling strategy captured transient morphogenetic events, including precise timing of cleavage (t2 to t8), onset of compaction, and rapid phenomena such as reverse cleavage or blastocyst collapse. Time-series image sequences were exported as digital video streams and synchronized image stacks for AI-driven morphokinetic parameter extraction.

### Dataset preparation and annotation

While continuous time-lapse imaging captured the entire developmental process up to the blastocyst stage, the dataset specifically prepared for the BE-DFNet instance segmentation model was strictly limited to the cleavage stages (from 1-cell to 16-cell/early uncompacted morula). A total of 847 high-quality images exclusively representing these stages were selected for annotation. Compacted morulae and later stages were deliberately excluded because, during compaction, blastomeres form tight junctions and exhibit high-density spatial overlapping. In 2D brightfield imaging, this obscures the physical boundaries of cells (especially on the opposite side of the equatorial plane), making individual cell counting and instance-level segmentation biologically ambiguous and physically unreliable. Each blastomere was pixel-wise annotated using LabelMe [21] by two experienced embryologists independently to generate ground-truth instance masks (Fig. 1c

**Fig. 1.**
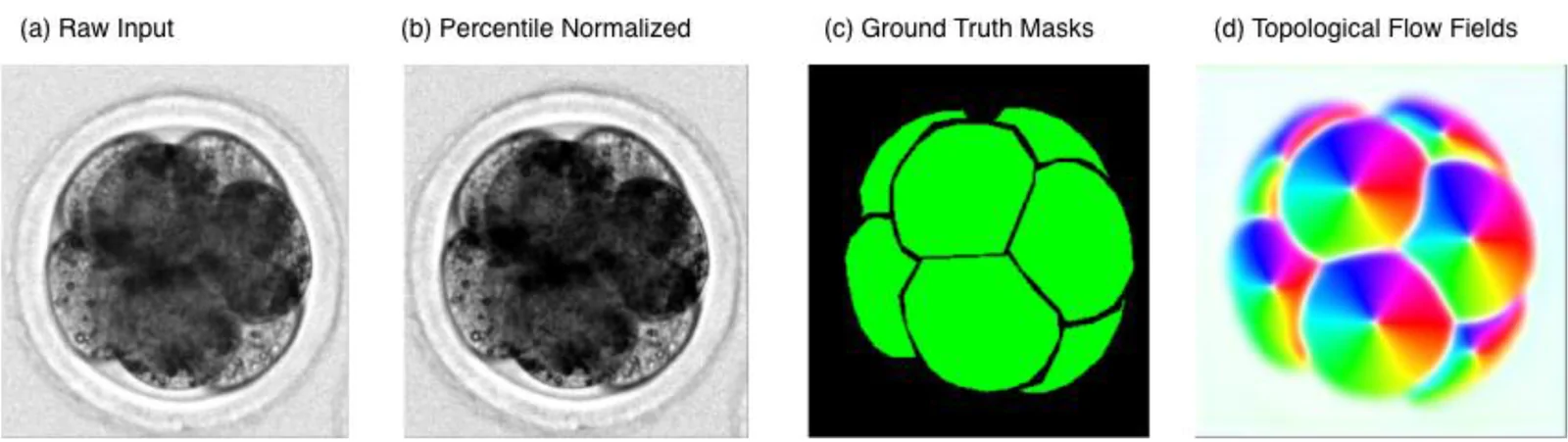
Workflow of data preprocessing and generation of supervision signals. (a) Raw microscopy image with low contrast and “dark-cell” artifacts caused by dense lipid granules. (b) Enhanced image after percentile normalization, showing clearer cell boundaries (enhanced input). (c) Manually annotated instance masks (ground truth). (d) Topological flow fields precomputed from ground-truth masks using heat diffusion dynamics; these vector fields serve as regression targets to enforce topological consistency.

); disagreements were resolved by consensus. To reduce texture interference from high-density lipid granules (“dark-cell” artifacts; Fig. 1a), a percentile-based intensity normalization preprocessing strategy was applied before training (Fig. 1b). Furthermore, topological flow fields were precomputed from these ground-truth masks using heat diffusion dynamics to serve as high-dimensional regression targets, thereby enforcing topological consistency during model training (Fig. 1d). To avoid leakage between training and testing, images were split by embryo at a ratio of 3:1:1 into training, validation, and test sets. All models were trained and evaluated using the same embryo-level split and identical preprocessing. 

### Architecture of the boundary-enhanced deep learning network

Given the “strong texture, weak boundary” characteristics of ovine embryo images, we designed a boundary-enhanced dual-flow fusion network (BE-DFNet; Fig. 2

**Fig. 2.**
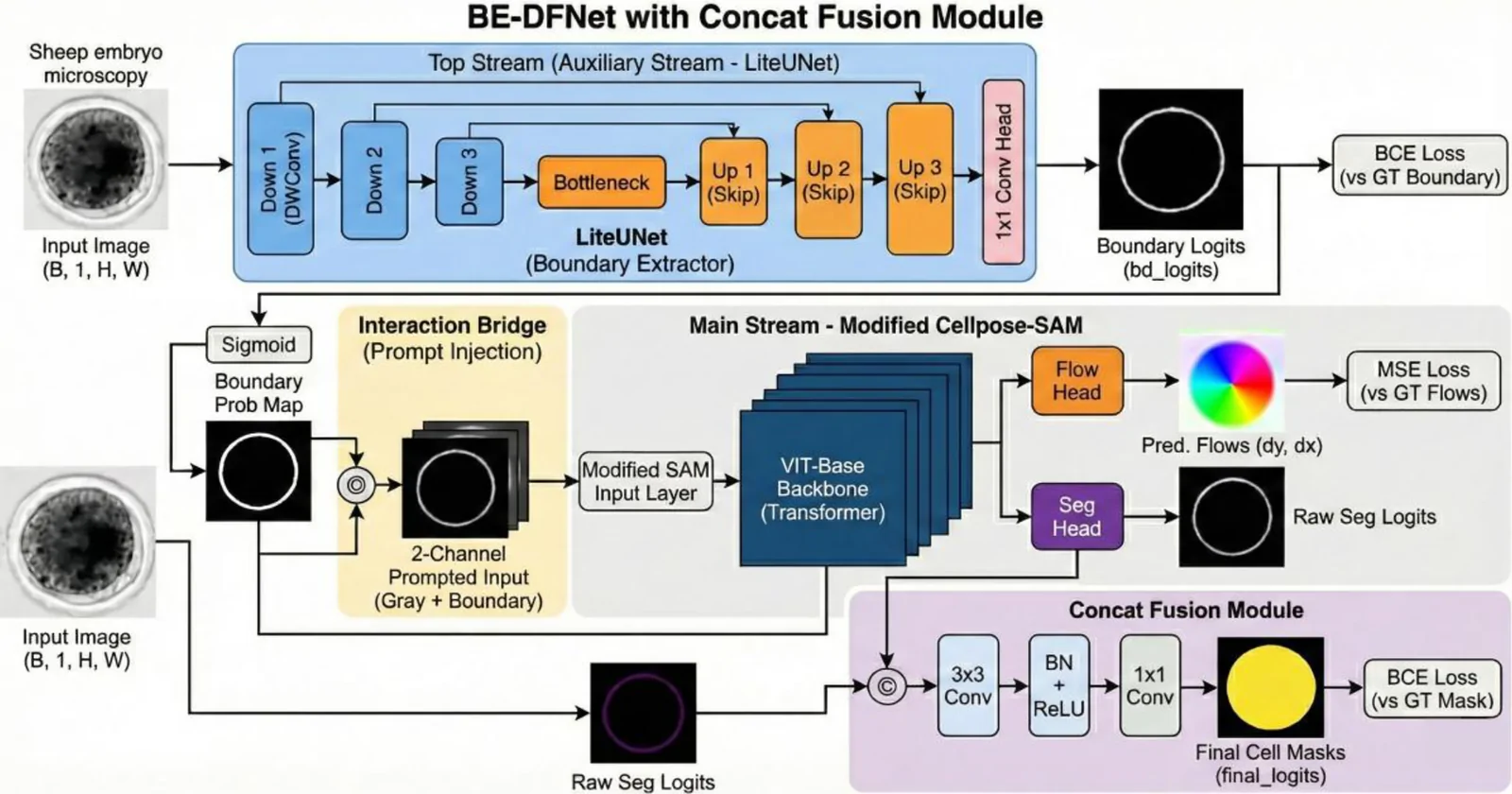
Overview of the BE-DFNet architecture.

). The network aims to mimic expert observation by simultaneously focusing on overall cell morphology and intercellular contact boundaries.

(1) Semantic stream: The main backbone extracts global morphological features using a ViT-based encoder to identify the main body of cells.

(2) Boundary stream: A lightweight auxiliary branch dedicated to detecting subtle physical contact interfaces (boundaries) between cells. With explicit supervision, this branch forces the network to attend to high-frequency edge information and suppress cytoplasmic texture noise in feature space.

(3) Dual-stream fusion: An asymmetric fusion mechanism injects structural information extracted by the boundary stream into the semantic stream. This design allows the model to leverage boundary cues in low-contrast regions, separating adhered cells and generating segmentation results with correct topology.

### Statistical analysis

Segmentation performance was evaluated using panoptic quality (PQ), endpoint error (EPE), and boundary intersection-over-union (Boundary IoU), among other metrics. EPE measures the discrepancy between predicted and ground-truth flow fields and is a key indicator of topological stability. Statistical analyses were performed using Python (SciPy library) [22]. Performance differences between models were assessed using Student’s *t*-test. P < 0.05 was considered statistically significant. All metrics were computed over the entire test set and reported as summary values.

## Results

### Quantitative evaluation and metric analysis

To facilitate understanding for the reproductive biology readership, the following evaluation metrics are defined: Dice coefficient measures the spatial overlap between predicted and ground-truth masks; Mean Average Precision (mAP) evaluates the precision across different overlap thresholds; and Aggregated Jaccard Index (AJI) accounts for both object-level and pixel-level segmentation accuracy by penalizing under- and over-segmentation.

Supplementary Table S1 summarizes segmentation results of BE-DFNet and baseline methods on the ovine embryo test set (170 images). BE-DFNet achieved a PQ of 0.5088, higher than Cellpose-SAM (0.4323). AJI increased from 0.5014 to 0.5425, mAP from 0.2968 to 0.3587, and Dice from 0.6679 to 0.7341 (Supplementary Table 1). Regarding flow-field consistency, EPE decreased from 1.6265 to 0.4632 (a 71.5% reduction; Supplementary Table 1). EPE was only computed for methods that output flow fields (Cellpose-SAM and BE-DFNet) and was not applicable to frameworks without flow-field outputs (Supplementary Table 1). In addition, BE-DFNet achieved a Boundary IoU of 0.2479, higher than Cellpose-SAM (0.1483), indicating improved separation at cell–cell contact boundaries (Supplementary Table 1).

Furthermore, we evaluated the computational efficiency of the models (Supplementary Table 1). The inference time of BE-DFNet was 28.8 ms per image, which is significantly lower than that of the baseline Cellpose-SAM (40.5 msec) and notably more efficient than other complex architectures such as TransUNet (82.1 msec). These results demonstrate that the boundary-enhanced dual-flow architecture achieves superior segmentation precision while maintaining high computational performance, making it suitable for real-time time-lapse monitoring.

### Computational efficiency and training convergence

Regarding the computational efficiency and training convergence (Fig. 3

**Fig. 3.**
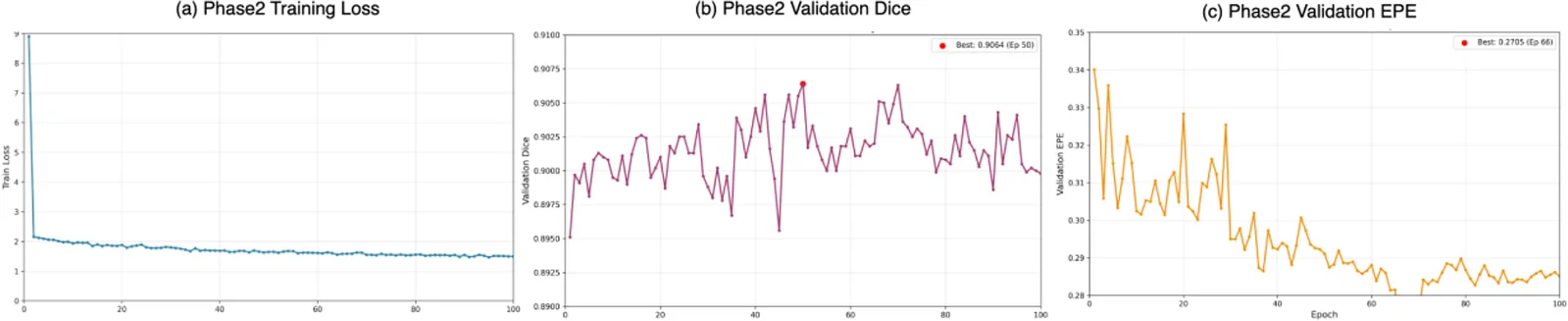
Training and validation dynamics of BE-DFNet. The curves illustrate the progression of training loss (a), the Dice coefficient on the validation set (b), and the Endpoint Error (EPE) on the validation set over 100 epochs (c). The synchronized decline in training loss and EPE, coupled with the steady increase in the Dice coefficient, demonstrates that the model effectively learns both topological flow and instance morphology, reaching stable convergence after approximately 75 epochs.

), BE-DFNet demonstrated high learning speed on the Sugon DCU K100 platform, with the total training time for Phase 2 being approximately 3.6 h. The training loss (Fig. 3a) and validation Endpoint Error (EPE) (Fig. 3c) exhibited a rapid, synchronized decline, indicating successful learning of boundary-flow representations. The validation Dice (Fig. 3b) coefficient reached 0.9064 within 50 epochs and remained stable thereafter, while EPE continued to refine until epoch 75 (Fig. 3). This synchronized decline demonstrates that the model effectively learns both topological flow and instance morphology during this phase, suggesting that the boundary-enhanced signals successfully guide the network to converge on a robust representation of blastomere structures.

### Stage-specific segmentation performance

Given that segmentation difficulty inherently increases with the number of blastomeres, we further performed a broad stage-specific evaluation (Supplementary Table 2). The BE-DFNet maintained a high level of accuracy during early cleavage (1- to 4-cell stages, PQ = 0.6734, Dice = 0.8546). As cell density increased at the 8-cell to 16-cell stages, segmentation difficulty naturally escalated due to tighter cell adhesion and overlapping boundaries. However, BE-DFNet still achieved reliable performance (PQ = 0.4126, Dice = 0.6718) at the 16-cell stage, successfully separating tightly packed blastomeres where conventional baseline models frequently failed. We further performed a broad stage‑specific evaluation (Supplementary Table 2), with 85, 122, 210, 260, and 170 images analyzed for the 1‑cell, 2‑cell, 4‑cell, 8‑cell, and 16‑cell stages, respectively. The distribution reflects the natural developmental frequency, with fewer early‑stage embryos (easy segmentation) and more mid‑stage embryos (challenging segmentation).

### Qualitative robustness across developmental stages

Figure 4

**Fig. 4.**
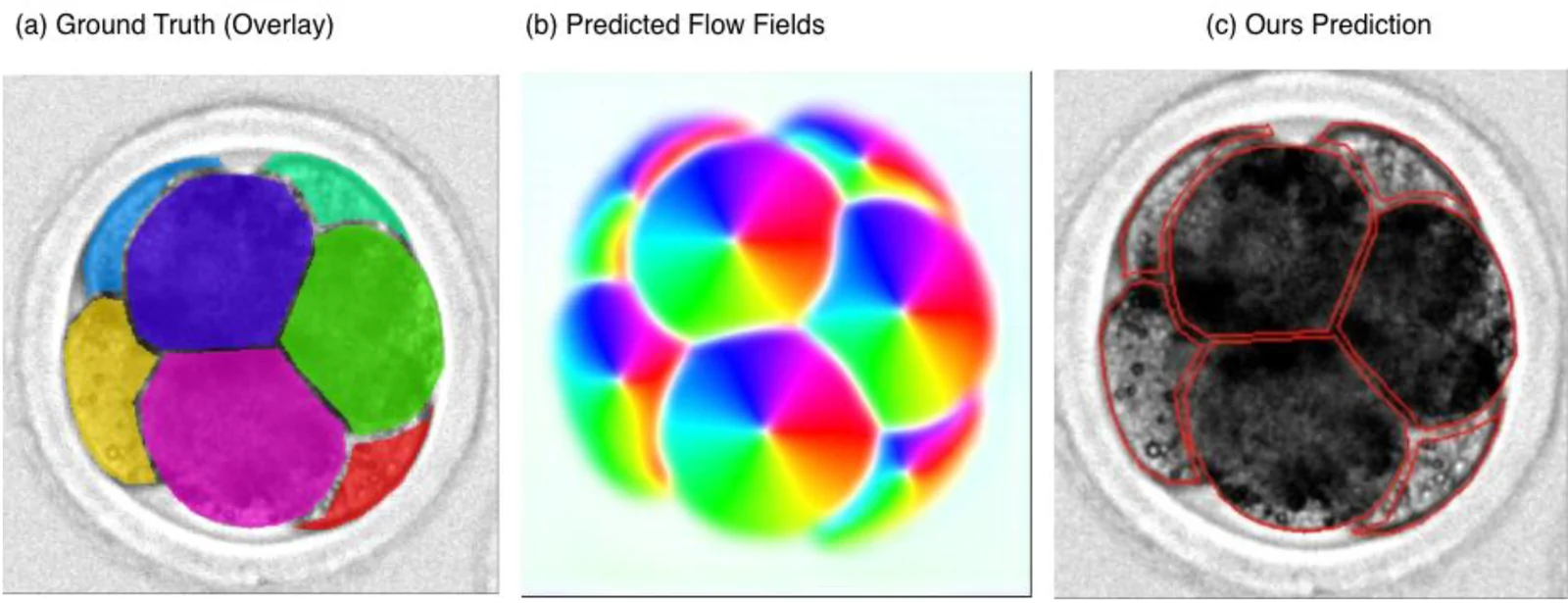
Qualitative segmentation results of BE-DFNet on a challenging ovine embryo sample with densely contacting blastomeres. (a) Ground-truth overlay (colored masks) on the raw image. (b) Predicted vector flow fields generated by BE-DFNet. Distinct color changes indicate that the model learned independent convergence centers for each blastomere, consistent with the low EPE score. (c) Final instance segmentation result (red contours), showing continuous boundary delineation at low-contrast contact interfaces, consistent with the improved Boundary IoU.

shows qualitative results on a representative ovine embryo sample with densely contacting blastomeres. The predicted flow field produced by BE-DFNet exhibited relatively independent convergence centers within each blastomere and clear separation at contact regions (Fig. 4b). The resulting instance contours were continuous along low-contrast interfaces (Fig. 4c), consistent with improved boundary performance. Specifically, the predicted flow field (Fig. 4b) exhibited distinct color changes within each blastomere, indicating that the model successfully learned independent convergence centers for individual cells. This sharp directional gradient at cell-cell interfaces is the mechanistic reason for the 71.5% reduction in EPE, as it prevents the ‘center-point drifting’ that typically causes instance merging in baseline models.

To further evaluate the robustness of BE-DFNet across early embryonic cleavage, we performed a qualitative comparison on embryos ranging from the 2-cell to 16-cell stages (Fig. 5

**Fig. 5.**
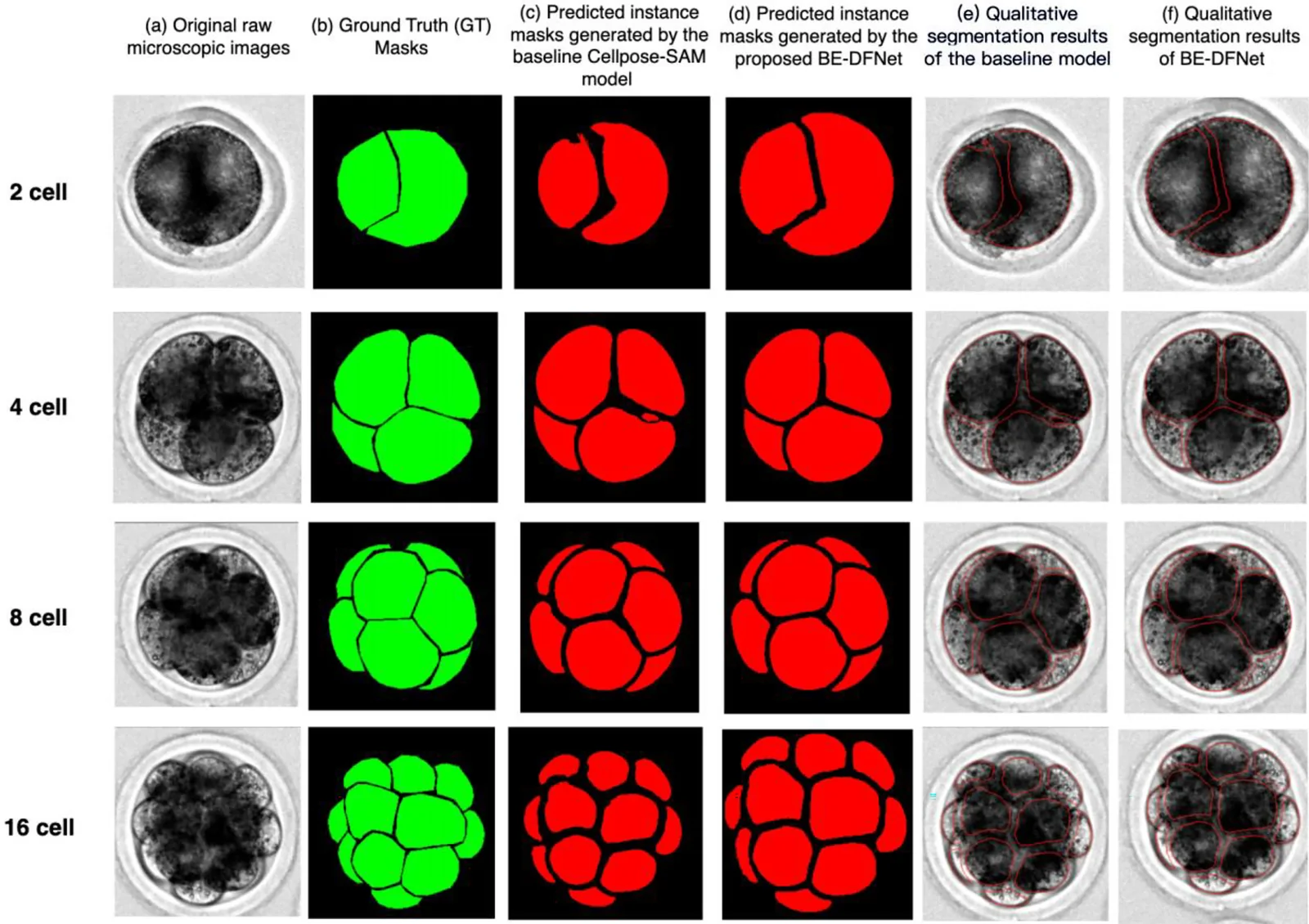
Visual comparison of segmentation performance across different embryonic developmental stages. Rows from top to bottom represent embryos at the 2-cell, 4-cell, 8-cell, and 16-cell stages. Columns from left to right show: (a) original images, (b) manual Ground Truth masks, (c) predicted masks by baseline, (d) predicted masks by BE-DFNet, (e) segmentation results of baseline, and (f) segmentation results of BE-DFNet. Red contours in (e) and (f) delineate the identified blastomere boundaries.

). As shown in the original microscopic images (Fig. 5a), the complexity of cell-cell adhesion increases significantly as development progresses from the 2-cell to the 16-cell stage. Compared with the manual Ground Truth (GT) masks (Fig. 5b), the baseline Cellpose-SAM model frequently suffers from under-segmentation in these high-density adhered regions. This is particularly evident at the 8-cell and 16-cell stages, where its predicted instance masks (Fig. 5c) and final segmentation contours (Fig. 5e) show clear merging errors, failing to distinguish individual blastomeres. In contrast, BE-DFNet consistently produces precise instance masks (Fig. 5d) and continuous boundary delineations (Fig. 5f) that align closely with the manual annotations. This visual evidence across multiple developmental stages confirms that our boundary-enhanced mechanism effectively captures morphological variations and maintains topological consistency throughout early cleavage.

### Attention map analysis of boundary-related responses

To investigate the mechanism of repair, we visualized attention heatmaps at the same intermediate layer (L11) for both models (Figs. 6a, b

**Fig. 6.**
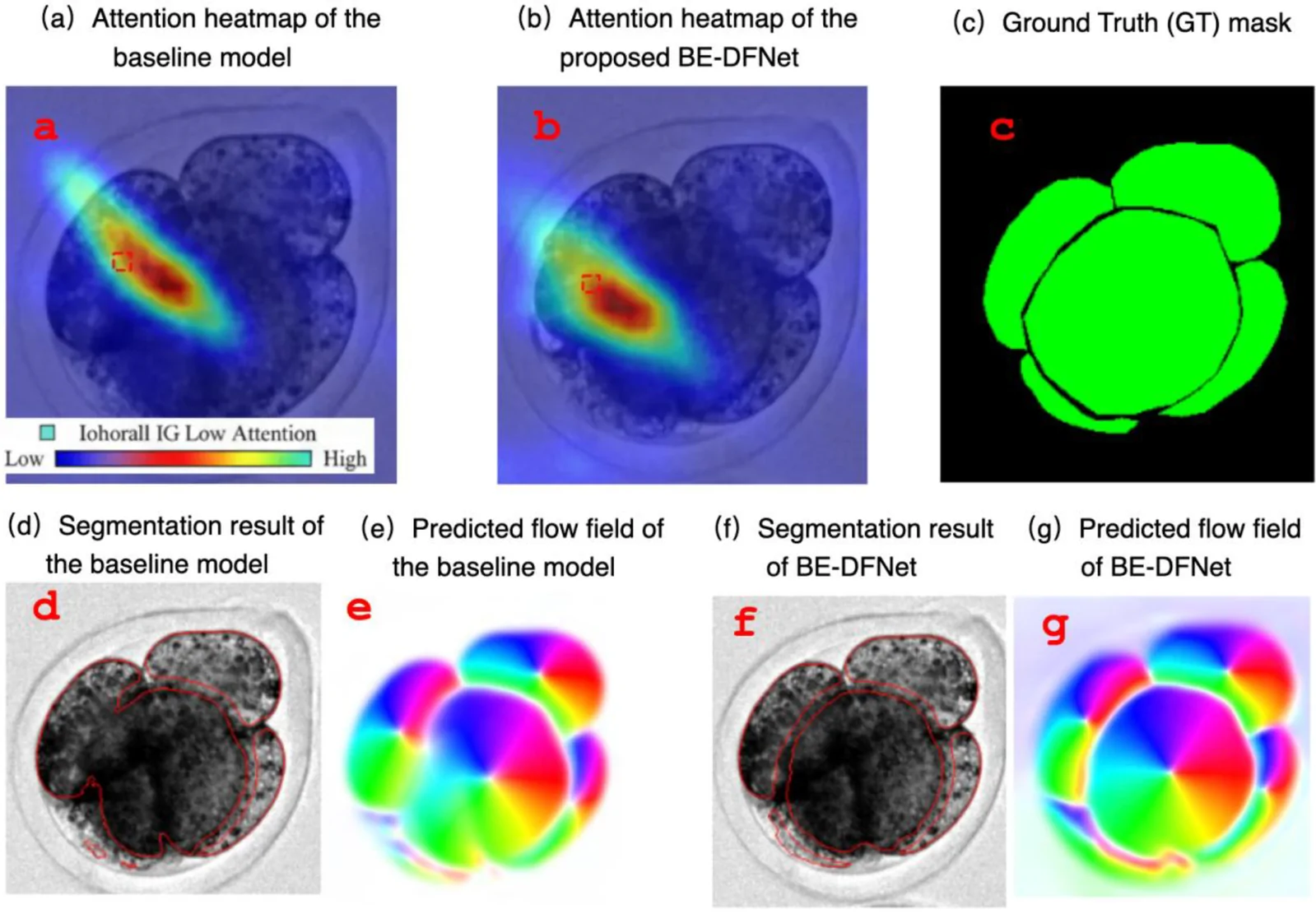
An XAI-diagnosis-driven closed loop for mechanistic repair. (a) Attention heatmap of the baseline model (Cellpose-SAM) at layer L11, showing diffuse attention focus that incorrectly extends significantly into neighboring cells, representing the root cause of under-segmentation. (b) Attention heatmap of BE-DFNet at the same layer (L11), demonstrating a precise focus strictly confined to the cell boundaries and their interior, without misleading expansion into the periphery. (c) Corresponding Ground Truth (GT) mask. (d) Qualitative segmentation result of the baseline model, showing merging errors. (e) Predicted flow field of the baseline model, displaying disordered vector directions. (f) Segmentation result of BE-DFNet (Ours), achieving precise separation. (g) Predicted flow field of BE-DFNet, exhibiting smooth and continuous vector directions.

). For the baseline Cellpose-SAM, the attention focus is markedly diffuse, extending significantly into neighboring cells (‘attention spillover’, Fig. 6a), which causes merging errors (Fig. 6d). In contrast, BE-DFNet’s attention is strictly localized within cell boundaries and the interior (Fig. 6b), effectively preventing misleading expansion and achieving precise individual cell recognition (Fig. 6f). This mechanistic repair is further evidenced by the predicted flow fields: while the baseline model displays disordered vector directions (Fig. 6e) that leak across boundaries, BE-DFNet produces smooth and continuous vector directions (Fig. 6g) strictly constrained by the boundary prior. These results visually confirm that the dual-stream fusion architecture successfully suppresses cytoplasmic texture noise and forces the network to prioritize high-frequency physical interfaces.

## Discussion

This study demonstrates that a boundary-enhanced dual-flow fusion network (BE-DFNet) substantially improves automated blastomere segmentation accuracy in ovine IVF embryos. To the best of our knowledge, this work is among the early efforts specifically addressing the instance segmentation challenges posed by ovine embryos’ high lipid content using deep learning. By explicitly integrating boundary information, the proposed model effectively overcomes the “strong texture, weak boundary” problem in ovine embryo images [18]. These findings support the potential of boundary-enhanced strategies for high-noise biological images and provide a technical basis for objective, automated embryo assessment.

Accurate determination of blastomere number and symmetry underpins the IETS grading system and prediction of implantation potential [7, 13]. Our results show that BE-DFNet reduced endpoint error (EPE) by 71.5% compared with the baseline model. This improvement is biologically meaningful because EPE directly reflects topological consistency. In practical applications, topological errors (e.g., merging two adjacent cells into one) can lead to undercounting and misclassification of developmental stage (e.g., mislabeling a 4-cell embryo as 3-cell) [15]. Such high-precision cell counting capability is a critical prerequisite for the automated extraction of morphokinetic parameters, such as cleavage timings (t2, t4, t8), and thus directly impacts the overall reliability of blastocyst development prediction. Previous studies indicate that blastomere number is a key morphokinetic parameter predicting blastocyst formation [8, 9]. Therefore, BE-DFNet’s improved segmentation can reduce misjudgment risk caused by counting errors and enhance embryo selection accuracy.

The mechanism for this improved performance was further elucidated via XAI-driven attention-map analysis. The “diffuse attention” observed in the baseline model may arise from the similarity between lipid granules and cell boundaries in texture features [23], making it difficult to distinguish cytoplasmic interiors from boundaries. Our comparison of attention heatmaps (Fig. 6) reveals that the baseline model suffers from significant ‘attention spillover’ into adjacent cells, which is the root cause of under-segmentation. By introducing a boundary stream as a structural prior, BE-DFNet effectively constrains the network’s attention to high-frequency physical interfaces, thereby suppressing interference from cytoplasmic texture noise. This is consistent with findings in human embryo studies showing that integrating multi-scale features helps identify embryonic structures under complex backgrounds [16].

In terms of model generalization, BE-DFNet exhibits robust performance across different experimental settings. By utilizing percentile-based intensity normalization and learning invariant boundary-flow representations, the model effectively mitigates the effects of varying illumination and magnifications (e.g., 200 × and 400 ×) common in different microscopic equipment. Furthermore, qualitative verification across the 2-cell to 16-cell stages (Fig. 5) demonstrates that the model maintains high precision as the complexity of cell adhesion increases during cleavage.

However, a methodological limitation of the current study is that the analysis was primarily performed on 2D brightfield images adjusted to the equatorial section. While this focal plane maximizes the visibility of most blastomeres, it may not fully capture cells located on the opposite side of the embryo without volumetric information. If blastomeres are entirely obscured, 2D-based instance segmentation may still face challenges in total cell counting. Accurate instance segmentation is only the first step toward automated embryo evaluation. Based on precise instance segmentation, key morphokinetic parameters (e.g., cell-cycle lengths t2, t4, t8 and blastomere size heterogeneity) can be extracted, and these have been associated with blastocyst quality and pregnancy outcomes in bovine and ovine embryos [9, 13, 24]. Future work will focus on integrating BE-DFNet into a complete 3D time-lapse analysis pipeline. Combining 3D imaging modalities (e.g., optical coherence tomography) may further improve comprehensive assessment given embryos’ three-dimensional structure [25].

A recognized limitation of the current BE-DFNet, and indeed of instance segmentation approaches in general, is its inapplicability to compacted morulae and blastocysts. Once an embryo reaches the compaction stage, the distinct blastomere boundaries are largely obscured, making the instance-level separation of individual cells unreliable. For these later developmental stages, the evaluation paradigm must shift from instance segmentation (cell counting) to semantic segmentation (assessing the macroscopic area ratios of the blastocoele, inner cell mass, and zona pellucida). The present BE-DFNet is exclusively designed to address the challenges of the cleavage stages, ensuring the accurate extraction of critical early morphokinetic events (e.g., t2, t4, t8). In conclusion, BE-DFNet is a segmentation tool optimized for ovine embryos with high lipid-associated texture. By reducing cytoplasmic texture interference, the method enables precise blastomere instance segmentation and topological reconstruction, offering an objective foundation for automated embryo selection in assisted reproduction.

## Conflict of interests

The authors declare no conflicts of interest.

## Supplementary Material

Supplement Files
