# Supplementary material for: Automated precise recognition of ovine embryo microscopic images using a boundary-enhanced deep learning network
Source: J Reprod Dev. 2026 Jun 21;72(5):786–93. doi: 10.1262/jrd.2026-024 (PMC13677194; doi:10.1262/jrd.2026-024)
Supplement: Supplementary file 1 [file jrd-72-5-786-s001.pdf]

Supplementary Table 1. Quantitative comparison on the ovine embryo dataset

Model	EPE↓	Bd IoU ↑	PQ↑	AJI↑	mAP↑	Dice↑	Inference time（msec）
U-Net	-	0.3544	0.4278	0.5025	0.3779	**0.8944**	**10.2**
Mask R-CNN	-	0.2009	0.2960	0.4458	0.1439	0.8075	58.5
TransUNet	-	**0.2938**	0.3439	0.4316	0.3250	0.9036	82.1
Cellpose-SAM	1.6265	0.1483	0.4323	0.5014	0.2968	0.6679	40.5
BE-DFNet	**0.4632**	0.2479	**0.5088**	**0.5425**	**0.3587**	0.7341	28.8[1]

[1] † Measured as the average processing time per 512×512 image on a Sugon DCU K100 (64GB VRAM) platform.

Supplementary Table 2. Stage-specific segmentation performance of BE-DFNet and baseline models across different developmental stages, with the number of images per stage

Developmental Stage	Number of images	Model	EPE	PQ	AJI	mAP	Dice	Bd IoU
1-cell	85	Cellpose-SAM	1.1247	0.6215	0.7236	0.5217	0.8014	0.1826
		BE-DFNet	**0.3215**	**0.7239**	**0.7852**	**0.5864**	**0.8947**	**0.2917**
2-cell	122	Cellpose-SAM	1.3582	0.5846	0.6819	0.4723	0.7732	0.1635
		BE-DFNet	**0.3864**	**0.6718**	**0.7431**	**0.5419**	**0.8563**	**0.2684**
4-cell	210	Cellpose-SAM	1.5731	0.5127	0.6124	0.3846	0.7245	0.1472
		BE-DFNet	**0.4217**	**0.6245**	**0.6926**	**0.4682**	**0.8127**	**0.2351**
8-cell	260	Cellpose-SAM	1.8254	0.3864	0.4725	0.2417	0.6321	0.1246
		BE-DFNet	**0.4852**	**0.4753**	**0.5538**	**0.3264**	**0.7146**	**0.2107**
16-cell	170	Cellpose-SAM	2.0176	0.2971	0.3846	0.1725	0.5428	0.0973
		BE-DFNet	**0.5219**	**0.4126**	**0.4917**	**0.2753**	**0.6718**	**0.1864**
